# An Electromagnetically Actuated Double-Sided Cell-Stretching Device for Mechanobiology Research

**DOI:** 10.3390/mi8080256

**Published:** 2017-08-22

**Authors:** Harshad Kamble, Raja Vadivelu, Mathew Barton, Kseniia Boriachek, Ahmed Munaz, Sungsu Park, Muhammad J. A. Shiddiky, Nam-Trung Nguyen

**Affiliations:** 1 QLD Micro- and Nanotechnology Centre, Nathan Campus, Griffith University, 170 Kessels Road, Brisbane, QLD 4111, Australia; harshad.kamble@griffithuni.edu.au (H.K.); munaz.ahmed@griffithuni.edu.au (A.M.); m.shiddiky@griffith.edu.au (M.J.A.S.); 2 School of Natural Sciences, Nathan Campus, Griffith University, 170 Kessels Road, Brisbane, QLD 4111, Australia; raja.vadivelu@griffithuni.edu.au (R.V.); kseniia.boriachek@griffithuni.edu.au (K.B.); 3 HealthTech-X, Menzies Health Institute Queensland, Griffith University, Gold Coast, QLD 4111, Australia; m.barton@griffith.edu.au; 4 Clem Jones Centre for Neurobiology and Stem Cell Research, Griffith University, Don Young Road, Nathan, QLD 4111, Australia; 5 School of Mechanical Engineering, Sungkyunkwan University, Suwon 16419, Korea; nanosingapore@gmail.com

**Keywords:** biomedical engineering, cell stretching, mechanobiology

## Abstract

Cellular response to mechanical stimuli is an integral part of cell homeostasis. The interaction of the extracellular matrix with the mechanical stress plays an important role in cytoskeleton organisation and cell alignment. Insights from the response can be utilised to develop cell culture methods that achieve predefined cell patterns, which are critical for tissue remodelling and cell therapy. We report the working principle, design, simulation, and characterisation of a novel electromagnetic cell stretching platform based on the double-sided axial stretching approach. The device is capable of introducing a cyclic and static strain pattern on a cell culture. The platform was tested with fibroblasts. The experimental results are consistent with the previously reported cytoskeleton reorganisation and cell reorientation induced by strain. Our observations suggest that the cell orientation is highly influenced by external mechanical cues. Cells reorganise their cytoskeletons to avoid external strain and to maintain intact extracellular matrix arrangements.

## 1. Introduction

The cells in a functioning multicellular system are continuously exposed to various mechanical forces. The ability of the cells to recognise these mechanical stimuli and transform them into chemical responses is known as a mechanotransduction [1,2,3]. It is well known that abnormalities in mechanotransduction signalling pathways affect the cell behaviour and tissue homeostasis, consequently leading to pathogenesis [4,5].

Mechanical forces related to the cyclic deformation of soft tissues are essential for the maintenance of various physiological conditions of organs such as the heart, blood vessels, and lungs [6]. The connective tissues of these organs contain a significant amount of fibroblastic cell types. The mechanotransduction mechanisms in fibroblasts are crucial to modulate tissue homeostasis [7]. Fibroblasts continually perceive external mechanical stimuli, which subsequently lead to the production and remodelling of extracellular matrix (ECM) components. For instance, numerous studies have reported that, under cyclic or static strain, fibroblast cells synthesise the ECM protein [8]. Fibroblasts anchor their actin cytoskeleton with ECM by linkage proteins called integrins. Integrins act as mechanosensors that sense the physical forces applied to the cell surface. The ECM-integrin-cytoskeleton integration plays a vital role in the functional and structural adaptation of cell in response to mechanical cues [9]. These mechanical signals are then transmitted to the cytoskeleton by the formation of an ECM-actin cytoskeleton linkage. This complex system promotes the assembly of focal adhesion and thereby induces the reconstruction of the actin cytoskeleton that is necessary for cell stiffening, gripping, and adherence.

The cytoskeleton reorganisation exerts adaptive responses in the cell under mechanical stimuli [10]. This process enables cells to regulate the formation of new lamellaipodia and to adjust their adhesion to resist physical deformation [11]. Thus, cell displacement has been observed for different amplitudes, types, and directions of mechanical strain. For instance, cyclic stretching at high frequency aligns the cells perpendicularly to the stretching direction [12]. In contrast, static stretching induces cells to align parallel to the stretching direction [13]. Hence, better understanding of the cellular response of the fibroblast culture, upon mechanical stimuli will provide an insight into examining how mechanical factors alter the physiology and behaviour of the fibroblasts. Thus, various techniques have been developed to introduce mechanical stimuli to the cellular microenvironment. Considering the complex in vivo microenvironment of the cells, the majority of the cell stretching approaches has been developed as in vitro platforms [14,15,16,17].

Most cell stretching approaches include the use of tweezers or micropipettes to induce the mechanical stimuli [18,19,20,21]. However, commercial cell stretching platforms such as Flexcell (Flexcell International Corporation, Burlington, NC, USA), Strex Systems for cell Stretching (STREX Inc., Osaka, Japan), and ElectroForce have been recently available [22,23,24]. Moreover, various customised stretching platforms have been reported in the last decade. The majority of these platforms utilise electromagnetic, pneumatic, piezoelectric, or optical actuators to deform an elastic membrane with cells cultured on it [14,25,26,27]. For instance, Shimizu et al. [28] developed a microfluidic device with serially connected balloons. The authors utilised a positive pressure to inflate the balloons and directly induce strain onto the cells. Similarly, Kamotani et al. [29] designed a microfluidic device with a deformable membrane at the bottom and an array of piezo electrically actuated pins, which were placed below the microwell plate. Each microwell was independently actuated by the pin, which deforms the micro-well membrane with cells cultured on it. Furthermore, Huang et al. [30] designed a cell stretching platform with an indenter and utilised a servomotor to introduce strain onto the deformable membrane. In another study, Sraj et al. [31] designed a microfluidic channel and utilised a single-mode laser (830 nm, 200 mW) to trap and deform the cells.

In addition to the above-mentioned actuation approaches, electrothermal, electrostatic, and dielectrophoretic actuations have recently been adapted to introduce mechanical force onto the cells [32,33,34]. All cell stretching approaches reported in the literature have their specific advantages. However, very few platforms provide the main features of a robust cell stretching tool such as a standardised strain pattern, a wide range of imaging options, and high-throughput capability. This paper presents a novel cell stretching platform with a double-sided uniaxial magnetically actuated stretching approach to introduce both homogeneous cyclic and static strain onto the cell culture. The cell stretching platform is homogenous and provides a wide range of strain values, cyclic and static stretching modes, compatibility with general clinical tools, and imaging options. Thus, our system is suitable for long-term cell stretching studies. The present paper discusses in detail the modelling, fabrication, and characterisation of the cell stretching platform and provides preliminary observations of the cellular response for fibroblasts under cyclic stretching, static stretching and non-stretching conditions.

## 2. Materials and Methods

### 2.1. Device Design and Working Principle

**Figure 1:**
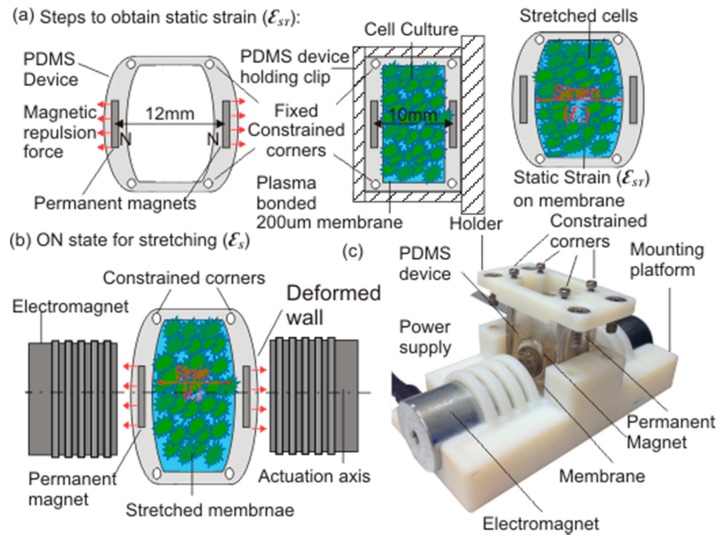
Schematic illustration of the working principle: (**a**) Steps to obtain static strain; (**b**) The ON state for cyclic stretching conditions; (**c**) Actual cyclic cell stretching platform and the polydimethylsiloxane (PDMS) device.

Figure 1 illustrates the schematic of the developed cell stretching platform. The platform consists of the polydimethylsiloxane (PDMS) device, with embedded permanent magnets, a holding clip for the static strain condition, and a mounting stage with electromagnets for cyclic strain conditions. The PDMS device has two NdFeB disc magnets (15 mm diameter and 2 mm thickness) embedded in the front and back wall, which are 4 mm thick and placed 8 mm apart.

The permanent magnets are positioned along the actuation axis such that the north poles of both magnets are facing each other to induce repulsive force and to deform the front and back wall of the PDMS device. This active magnetic repulsion was utilised to introduce the static strain onto the deformable membrane. The custom made 3D printed holding clip was used to overcome the active magnetic repulsive force and to maintain the PDMS device in a predefined position. The 200-µm thick deformable membrane was then bonded at the bottom of the device after oxygen plasma treatment. Removing the holding clip results in the desired static strain on cells cultured on the deformable membrane, Figure 1a. This simple but effective strategy allows a wide range of predefined strain on the deformable membrane to be achieved, simply by controlling the initial and final position of the holding clip during the bonding process of the deformable membrane and the PDMS body.

For cyclic strain, two axially aligned electromagnets (JL Magnet, JL Magnet Co., Ltd., Seoul, Korea) controlled by a DC power supply (MK Power, M-K Power Products Corporation, Mississauga, ON, Canada) were used simultaneously to externally actuate the magnets embedded in the PDMS device. The mounting platform was 3D printed to introduce the necessary constraint onto the PDMS device and to provide axial alignment for the permanent magnets (PMs) and electromagnets (EMs). Upon actuation, the magnetic forces generated by the EMs and the PMs, deforms the front and the back wall of the PDMS device. This force is further transferred to the deformable membrane, which in turn induces a well-defined strain onto the cells cultured there, as in Figure 1b. Figure 1c shows the actual developed cell stretching platform, which is capable of inducing homogeneous cyclic strain onto cells cultured on a deformable membrane, for studying the behaviour of the cells.

### 2.2. Modelling and Fabrication

A finite element analysis (FEA) model of the PDMS device was formulated in COMSOL Multiphysics 5.2 (COMSOL, Inc., Burlington, MA, USA) to understand and to optimise the stretching device. We modified and updated the previously reported FEA model to achieve the necessary parametric optimisation of the device [35]. The grade of the permanent magnets (PMs) was the key optimisation parameter, which was taken into consideration in order to manipulate the active magnetic repulsive force to obtain optimised static strain conditions for the PDMS device without physically damaging the device.

To start with, a reference FEA model was formulated in COMSOL to understand the magnetic field requirement. The two PMs were defined at the axial distance of 8 mm, and the diameter and thickness of the PMs were defined as 15 mm and 2 mm, respectively. The NdFeB material was assigned for the adapted geometry, and the PMs were modelled using Maxwell-Amperes law.

(1) ∇ × H = J, B = ∇ × A, J = σ E B

where **B** is the magnetic flux density in T, **E** is an electric field in V/m, A is magnetic vector, and **H** is the magnetic field strength in A/m.

**Figure 2:**
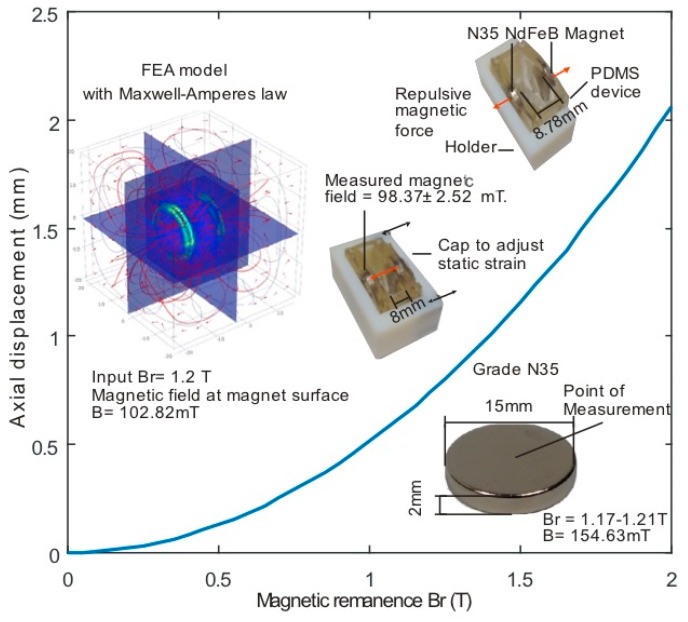
Simulated axial displacement versus magnetic remanence. (Inset: finite element analysis (FEA) results with Br = 1.2 T, selected NdFeB grade N35 magnet, PDMS device with static strain condition).

Furthermore, the optimisation was carried out by varying one parameter while maintaining the other parameters constant. Maintaining the geometrical parameters constant, the magnetic remanence (Br) of the PMs was varied over the range of 0.05 T to 2 T, with an incremental step of 0.05 T, using a parametric sweep function to model the variation of the NdFeB magnet grade. The inset in Figure 2 shows the results for the FEA model with an input magnetic remanence (Br) of 1.2 T. In the next step, to measure the corresponding deformation, the FEA model geometry of the PDMS device was built with a front and back wall thickness of 4 mm, a side wall thickness of 2 mm, deformable membrane thickness of 0.2 mm, and axially aligned PMs with 15 mm diameters and 2 mm thickness embedded into the front and back wall. The total volume of the geometry formulated in COMSOL was 30 mm × 25 mm × 12 mm.

Next, the study of the structural mechanics and the magnetic force was coupled in COMSOL to estimate the outward force acting on the front and back wall for various values of magnetic remanence. The appropriate material properties (750 kPa Young’s modulus and 0.49 Poisson’s ratio for 10:1 PDMS-cross linker mixture) were selected to match the real device [36,37,38]. All four corners of the device were fixed to maintain the necessary boundary conditions. Figure 2 shows the average displacement versus the magnetic remanence of the PMs along the actuation axis. The parametric optimisation suggested that 1.2 T magnetic remanence (grade N35) yields a magnetic flux of 103 mT at the surface of the PM and generated an average displacement of 0.788 mm between the two embedded PMs along the actuation axis. Thus, for the static condition, the magnetic repulsion facilitated a maximum static strain of 9.85% onto the membrane. Considering the experimental requirement, a NdFeB magnet of grade N35 was selected for the cell stretching system.

The next step was the fabrication of the optimised design. The fabrication process involved the fabrication of a master mould, a mounting platform, and the PDMS device. To replicate the PDMS device with the optimised geometry, all parts of the master mould were designed with the necessary fabrication tolerance using SolidWorks 2013 (Dassault Systemes Solidworks Corp., Waltham, MA, USA). Each part was then carefully cut from the non-magnetic aluminium material and milled as per the design specifications. Finally the necessary screwing holes were drilled, and the parts were assembled to obtain the master mould.

The mounting platform and holding clip were also designed in SolidWorks 2013 to achieve the necessary constraint and axial alignments. The optimised mounting platform and holding clip designs were 3D printed using an Eden 260 V printer (Stratasys Ltd., Eden Prairie, MN, USA). In the final step, the PDMS device and the deformable 200 μm membrane were fabricated.

For the fabrication of the PDMS device, 20 g of degassed mixture of PDMS and cross linker (Sylgard 184 elastomer kit, Dow Corning, Midland, MI, USA) was prepared with a volume ratio of 10:1. The mixture was poured into the master mould and was again degassed for 15 min to remove any remaining air bubbles. The mould was then carefully closed to cure the PDMS mixture for 2 h at 80 °C in a vacuum oven. Once cured, the mould was carefully opened, and the PMs were placed into the created cavities such that the north poles of both magnets faced each other in order to achieve active magnetic repulsion condition. To fix the magnets in position, a small amount of PDMS-cross linker mixture (10:1 volume ratio) was coated onto the PMs. The mould was then carefully closed and cured for another 30 min at 80 °C in a vacuum oven to ensure the proper placement of the PMs. The cured PDMS device was then inspected and carefully removed from the mould. Finally, the PDMS device was cleaned with isopropanol and de-ionized (DI) water. In the next step, a degassed PDMS-cross linker mixture with a volume ratio of 10:1 was spin coated at 400 rpm for two min and cured at 80 °C for two hours to achieve the 0.2-mm thick deformable membrane [39,40]. The cured membrane was inspected to confirm its uniform thickness and then cleaned with isopropanol and DI water. In the last step, the PDMS device was plasma bonded with the deformable membrane and cured for one hour at 80 °C.

### 2.3. Cell Culture

Mouse 3T3 fibroblast cells were cultured in DMEM/F12 (Gibco, Thermo Fisher Scientific, Waltham, MA, USA) medium with 10% fetal bovine serum (FBS) and 1% penicillin at 37 °C and 5% CO_2_ in a standard incubator. To sterilise the device, the device was treated with 80% ethanol and washed three times with 1× phosphate buffered saline (PBS) followed by ultraviolet (UV) exposure for 20 min. Before seeding the cells, the device was treated with fresh media and incubated for one hour under standard conditions (37 °C and 5% CO_2_) to further ensure its biocompatibility. For seeding the device, 80% confluence was reached in T75 flask and the cells were harvested at the optimised density of 75 × 10^3^ Cells/250 μL. In order to achieve the optimal adherence and growth of the cells on the membrane, the device was incubated at 37 °C and 5% CO_2_ for 24 h before the mechanical strain was applied.

### 2.4. Application of Strain on Fibroblasts

Two stretching modes were tested, namely, cyclic and static axial stretching, and compared to the non-stretching (control) condition for this study. Cyclic stretching was applied to the cells with 1.4% strain at 0.01 Hz and 50% duty cycle. For a comparative study, the same 1.4% strain was applied to the fibroblast cell culture under the static condition. The strain was introduced onto the cells over five different time instances (0.5 h, 1 h, 2 h, 3 h, and 4 h) for a maximum of 4 h. The cells’ characteristics such as area, aspect ratio, and orientation were observed after stretching and compared to the control. For the analysis, the central region of the membrane was imaged with an inverted microscope (Nikon Eclipse Ts2, Nikon Corporation, Tokyo, Japan). Biological triplicates were performed.

### 2.5. Cell Fixing, Immunofluorescence Staining and Imaging

The stretched cells were fixed with 4% paraformaldehyde (PFA) for 15 min followed by the three 10-min PBS washes. The deformable membranes with fixed cells were stored in the PBS solution at 4 °C. For actin and nuclei observation, the cells were incubated with ActinGreen^TM^ 488 and NucBlue^TM^ ReadyProbe^TM^ Reagent (Thermo Fisher Scientific) for 30 min, followed by three post-staining washes with PBS. Images of the cell nuclei and actin fibres were finally obtained with a fluorescent microscope (Olympus BX50, Olympus Corporation, Tokyo, Japan) using 10× and 20× magnification.

### 2.6. Image Analysis

For the image analysis, three separate locations within the central region, i.e., the region of interest (ROI) with homogenous strain, were captured. Each image was enhanced using post processing with ImageJ (National Institutes of Health (NIH), Bethesda, MD, USA), which mainly included fast fourier transform (FFT) bandpass filtering, sharpening, enhancing the image contrast, and thresholding. For quantification, the captured cells were analysed to estimate the averaged area, aspect ratio, and orientation of the cells in ROI at each time instance.

## 3. Results and Discussion

### 3.1. Force Calculation

For estimating the experimental magnetic force, we utilised a similar approach as that reported in our previous work [41]. The optimised FEA model of the PDMS device was utilised to obtain the spring constant for the experimental conditions. Considering the stretching condition, the magnetic actuation was modelled in COMSOL by introducing an outward force onto the PMs to deform the front and back wall of the PDMS device. Further, simulated force input within a FEA model was varied from 0.5 N to 1 N with an increment of 0.5 mN for each step, and the corresponding displacement of the PMs was obtained for each point. Considering the material properties, we assumed the Hooks law to obtain the spring constant *k* for this study:

(2) F=−k·x,

where, *k* is the spring constant in N/mm, *F* is the force in N, and *x* in the displacement in mm.

The next step was to experimentally obtain the displacement of the PMs over the applied voltage. Both EMs were simultaneously actuated by supplying voltage ranging from 1 V to 30 V. The corresponding displacement of the marked points on the PDMS device wall along the actuation axis was recorded for each step using a digital camera (EO Edmund Optics, Edmund Optics, Barrington, NJ, USA). Furthermore, our particle tracking algorithm based on digital image correlation and the Matlab image processing toolbox was utilised to detect and measure the displacement of the randomly marked points [41]. Finally, the obtained average displacement was used to calculate the force using a spring constant of 2.41 N/mm, determined by the FEA simulation.

**Figure 3:**
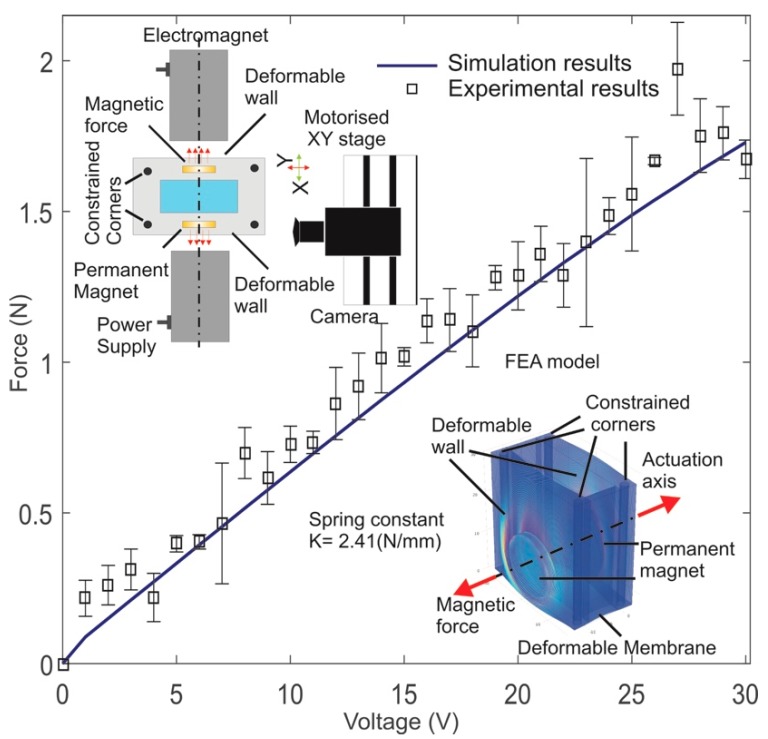
Magnetic force over the voltage range of 1 V to 30 V (Inset: Experimental setup and FEA model for PDMS device).

In the next step, we modified and updated our previously reported FEA model to calculate the magnetic force between the PM and the EM [35] and to validate the experimental data. We considered the symmetric nature of the system and obtained the magnetic force at the PM surface along the actuation axis over the voltage range of 1 V to 30 V [35]. The simulation results were verified with the experimental data in Figure 3. As expected, a linear force-voltage relationship can be clearly observed from Figure 3. The simulation agrees well with the experimental data. The results provide an acceptable error variance of 9.42% over the range of 9 V to 30 V between the experimental and simulation data.

### 3.2. Strain Calculation

**Figure 4:**
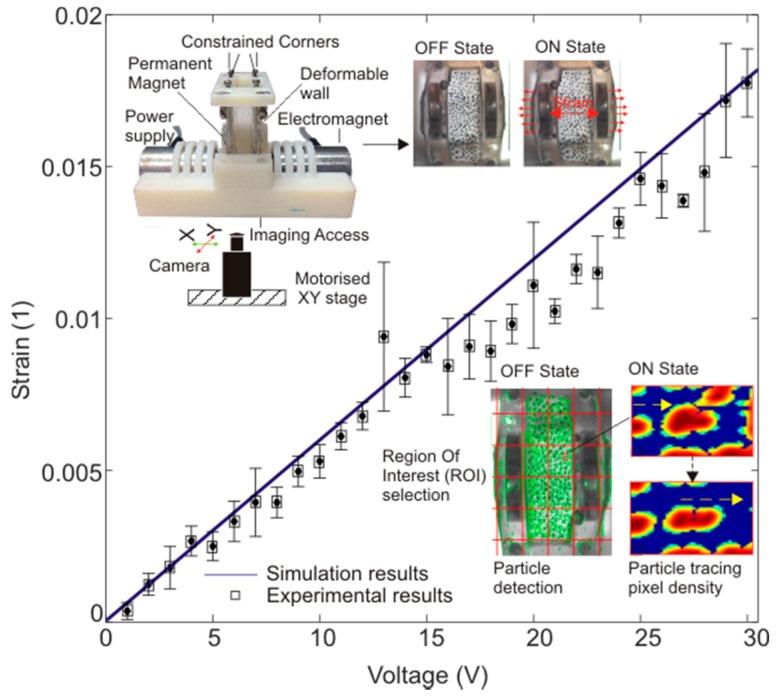
Strain on the deformable membrane over the voltage range of 1 V to 30 V. (Inset: experimental arrangement, the membrane in an ON and OFF state, an example of particle detection and tracking).

The characterisation of the strain applied to the deformable membrane was observed using both experiments and simulation. For measuring the strain experimentally, the membrane deformation was recorded with a digital camera (EO Edmund) over the voltage range of 1 V to 30 V. The particle detection and displacement measurement algorithm based on digital image correlation and the Matlab image processing toolbox was further utilised to calculate the offset displacement of the marked points. For reliable experimental data, the membrane of each recorded image was divided into 2 × 5 regions. A minimum of three marked samples from the central region (M_1,2_, M_1,3_, M_2,2_, M_2,3_) was observed. Finally, to warrant the repeatability of the results, three experimentally obtained results were averaged to represent the displacement of the region. The inset of Figure 4 depicts the experimental setup and an example of the particle detection and tracking algorithm result.

For cross validating the experimental data, we utilised a reference FEA model. The magnetic force obtained from the force calculation (Section 3.1) over the voltage range of 1 V to 30 V was used as the input for the FEA model. The central region of the membrane was considered the region of interest (ROI).

An average strain across the membrane was obtained for the operating voltage range, i.e., 1 V to 30 V. Figure 2 compares the average strain over the ROI from both the simulation and the experiments. The experimental and simulation results agree well. An average error variance of 7.89% was observed over the voltage range of 9 V to 30 V. Based on the strain characterisation, we selected an input voltage of 27 V for both actuators, which provided an average homogeneous cyclic strain of 1.38 ± 0.021% over the central region of the membrane.

**Figure 5:**
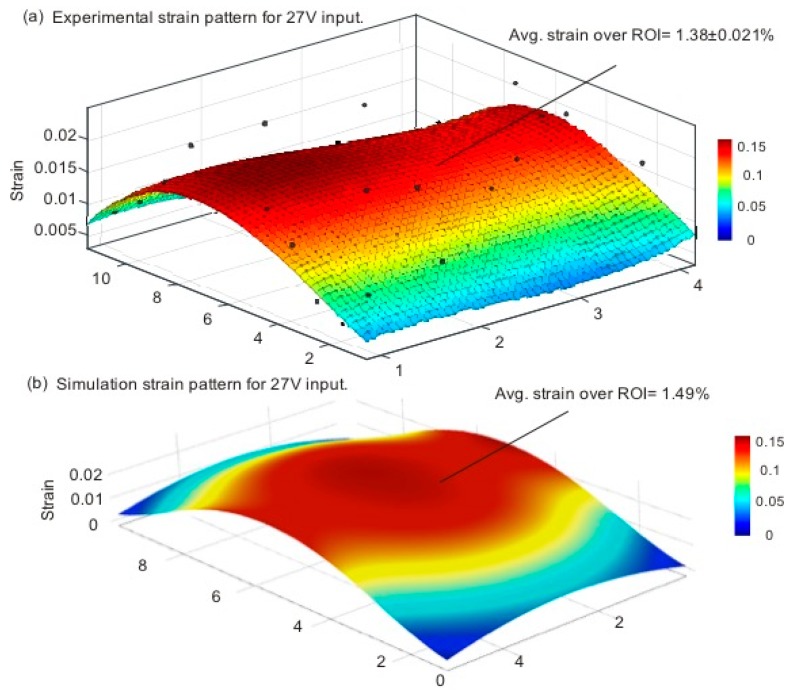
Strain pattern on the membrane with a selected input of 27 V: (**a**) Experimental results; (**b**) Simulation results.

For an understanding of the membrane deformation and strain pattern with the selected input voltage of 27 V, we utilised the same experimental platform and obtained the image sequence for the membrane deformation. The images were analysed using the existing particle detection and tracking algorithm to obtain the strain pattern over the 2 × 5 region matrix. A minimum of three marked points from each subregion was evaluated to obtain reliable results. Finally, the average value was utilised to represent the strain over each predefined subregion. Furthermore, three experiments were conducted for each set of data. Figure 5 shows the obtained strain deformation pattern from (a) the experiment and (b) the simulation. The expected, homogenous strain pattern over the central region of the membrane is evident from the results. The experiment and the simulation agree well and provide an average strain of 1.38 ± 0.021% and 1.49%, respectively.

The results provide a better understanding of the membrane deformation and confirm the homogenous strain pattern for the subsequent cell stretching experiments. Figure 5 clearly shows that we could expect a homogenous strain pattern in the central region of the membrane. Thus it can be assumed that cells located in this region will experience an equal amount of strain.

### 3.3. Cell Area and Aspect Ratio

**Figure 6:**
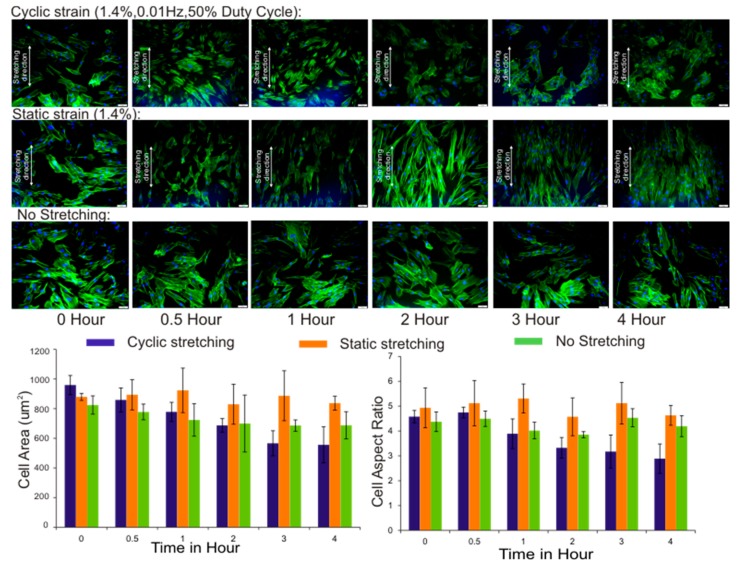
Analysis results of the cell area and aspect ratio for cyclic, static, and no stretch conditions over the predefined time points (Inset: fluorescent images of the fibroblast cells with a 20× objective for the corresponding time points with a 50-µm scale bar).

Figure 6 shows the obtained averaged cell area and aspect ratio for each predefined time point. The results show the gradual decrease in the cell area under cyclic stretching as a result of cell displacement and aggregation. The cell aspect ratio over the stretching duration for a maximum of 4 h also decreased, which was in line with the previously reported observations [42,43,44,45]. The morphological observation suggests that both cyclic and static stretching led to significant changes in fibroblast adherence. However, we focused more on cyclic stretching, as native tissues within the body are more exposed to cyclic strain rather than static strain. Under cyclic strain, mechanotransduction and intercellular physiology involving cytoskeletal elements and adhesion molecules drive the morphogenetic process of cells [46]. The cells respond adaptively against external stress transmission and reorganize their cytoskeleton integrity by reconstructing the actin stress fibres [47,48]. We also observed that cyclic stretching of the fibroblast cell culture triggered the formation stress fibres, and over time cells enhanced their cell-cell connection and formed cell clusters.

The reorganization of actin stress fibres seems to promote a cell adhesion dynamic and changes in cell morphology [49]. Furthermore, it was interesting to note that, after two hours of cyclic stretching, significant cell cluster formation was observed. The stronger cell connections and the formation of cell clusters explain how individual cells sense and transmit physical forces to and from neighbouring cells, that is, by the binding of adhesion molecules to expand cell-cell cohesion [50].

Furthermore, it is interesting to address the question as to why stretching induces cell rearrangement and the adhesion of the cells into clusters. Generally, cells on a substrate surface are dynamic in nature and are constantly reorganizing their actin filament network to retract and extend protrusions (the formation of lamellipodia and filopodia). At the cellular level, changes in the surface topology lead to the underlying processes involved in the maintenance of mechanical homeostasis [51]. The stress fibres are projecting tension-bearing bundles of actin filaments, which act as non-muscle sarcomeres [52]. Cyclic stretching seems to trigger actin fibres to realign and to establish stronger adhesive cell-cell connections to resist deformation [53]. Thus we hypothesise that actin organization under stress is critical for promoting cell adhesion dynamics to form cell aggregates and withstand the applied strain.

In contrast, the cell area and aspect ratio with static stretching did not change significantly. The cells did not spread and promoted the formation of actin stress fibres under static strain as compared with cyclic strain, as in Figure 6. Additionally, no significant cell clusters were observed in the static strain mode. This discrepancy further suggests that prolonged static stretching imposes different effects on the regulation of ECM and adhesion proteins. Previous investigations into the effect of static strains alone on ECM synthesis showed the degradation of ECM and adhesion molecules [54]. In a recent study, Cui et al. [55] showed that cytoskeleton organization differs for cyclic and static and indicated that cyclic stretching promotes actin fibre formation and cell spreading.

Moreover, as expected for non-stretching (control) conditions, the actin stress fibres were randomly distributed and no significant changes in the cell area and aspect ratio were observed.

### 3.4. Cell Orientation

**Figure 7:**
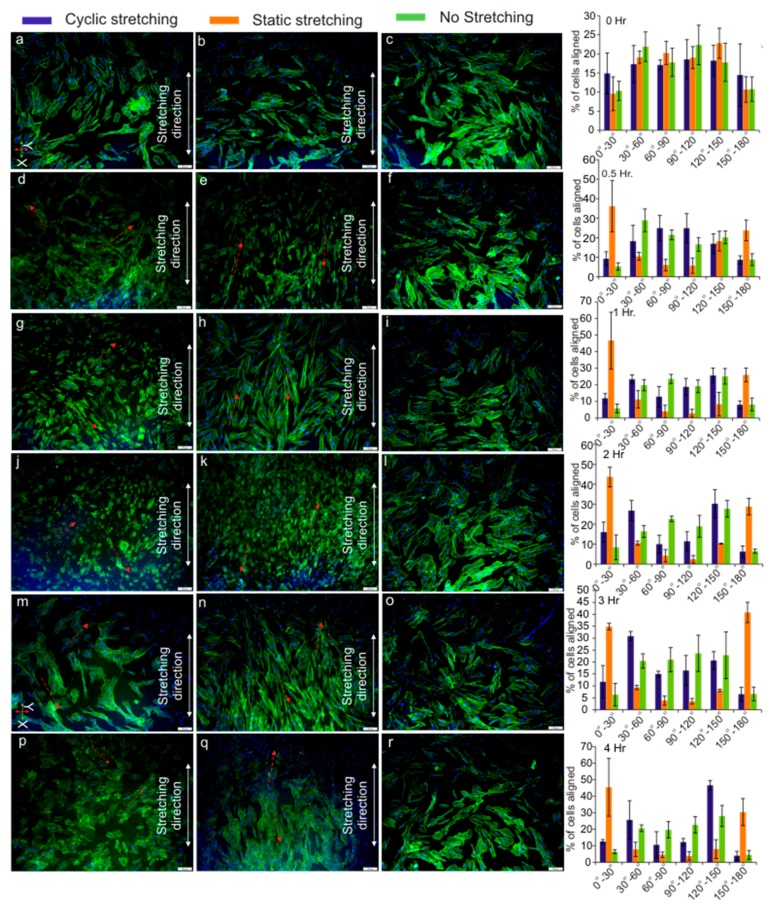
Cell orientation analysis results for cyclic stretching (1.4%, 0.01 Hz, 50% Duty Cycle), static stretching (1.4%), and no stretching conditions at 0 h, 0.5 h,1 h, 2 h, 3 h, and 4 h. (Inset: fluorescent images of the fibroblast cells with 10× objective for (**a**–**c**) 0 h; (**d**–**f**) 0.5 h; (**g**–**i**) 1 h; (**j**–**l**) 2 h; (**m**,**n**) 3 h; and (**p**–**r**) 4 h with 100-µm scale bar).

The orientation range of 0° to 180° was considered for the cell orientation analysis, which was further divided into six equal angular regions in 30° increments. For the quantitative analysis of the cell orientation, the 0° to 180° range was set along the stretching direction in an anti-clockwise direction, and a total of 900 samples from the three images of the ROI were analysed using ImageJ for each time instance. Figure 7 shows the distribution of the cell orientation for each time instance. It was interesting to observe distinct cell orientation trends for the cyclic and static stretching modes. Under the cyclic stretching mode, the cell orientation showed two distinct peaks over the 30° to 60° and the 120° to 150° ranges. Prominent cell orientation was observed after 1 h of cyclic stretching, whereas, under the static stretching conditions, two distinct peaks were observed over the 0° to 30° and the 150° to 180° ranges. Furthermore, it was interesting to observe that the prominence of the cell orientation under the static stretching conditions increased over the time. From this observation, we can conclude that most of the cells are oriented approximately either at 45° or 135° under cyclic stretching and at 15° or 165° under static stretching conditions (Figure 7 inset depicted by arrows).

Recently, Ugolini et al. [56] reported the ability of human primary cardiac fibroblasts to differentiate between cyclic mechanical stimuli and controlled oxygen tension. Moreover, in another study, Ugolini et al. [57] subjected human primary cardiac fibroblasts (CFs) to 2% and 8% cyclic strain for 24 and 72 h and interestingly observed the different cellular responses of the CFs based on the duration and amplitude of strain. In the present study, we observed a similar trend. Under a constant strain of 1.4% and duration of 4 h with the same culture conditions, we observed different cellular responses for the static and cyclic modes. Furthermore, we also observed that, under static strain, fibroblasts aligned parallel to the stretch direction, while fibroblast cells reoriented approximately ±45° to the stretching direction under 1.4% cyclic strain for 4 h, which was in line with the above observations. We hypothesise that, with further parametric optimisation of the amplitude and duration of the applied strain, the widely accepted perpendicular alignment could be achieved for the cyclic stretching mode. Overall, our observation further strengthens the argument that cells are capable of differentiating, not only between different types, but also between different magnitudes of stimuli.

Furthermore, as expected for the non-stretching conditions, a random distribution of the cell orientation was observed. Two distinct and almost symmetric peaks were observed for both the cyclic and static stretching modes, which provide important evidence on the ability of the fibroblast cells to recognize and respond not only to the applied strain but also to the strain direction [58,59,60]. Moreover, the arrangement of actin stress fibres is clearly involved in the realignment of the cells under strain. Our observation agrees well with previous studies, which reported that cells reorient and align themselves due to external strain [58,61,62,63,64,65].

The adhesion dynamics of cells are guided by cytoskeleton rearrangement and were believed to be responsible for cell alignment [53,59]. The cell–ECM connections are established by association between actin stress fibres and the focal adhesion, which endows cells to form stronger ECM connections and cell-cell adhesion [66,67]. The focal adhesion is responsible for reorienting cells in a direction in which cells can maintain their stability [59]. In line with this hypothesis, we also observed cytoskeleton organisation and a distinct cell orientation under both cyclic and static stretching conditions. In contrast, the non-stretching (control) conditions led to a random distribution of the cell orientation.

## 4. Conclusions

In summary, we developed a simple yet effective cell stretching platform capable of introducing homogeneous cyclic and static strain onto the cell culture. The characterisation of the developed platform provides a clear understanding of the device function. Furthermore, the effectiveness of the developed platform was tested with stretching assays of fibroblasts. Our preliminary analysis suggested that the orientation of the cells is highly influenced by external mechanical cues. The cell aggregation observed in the cyclic stretching mode suggests that the cells reorganise their cytoskeleton to avoid external strain and to maintain intact their extracellular matrix arrangements. The developed cell stretching platform may serve as a tool to investigate the cell behaviour under a wide range of strain with cyclic or static stretching modes. Moreover, considering these preliminary results, the platform may facilitate the active manipulation of fibroblasts to achieve the desired cell arrangements. This alternative cell culture method will have a broad range of applications in the field of tissue remodelling and regenerative medicine. Furthermore, it is also important to note that cells can be harvested after stretching using general clinical tools to perform standard biology analysis, which could be critical for clinical diagnosis and subsequent therapeutic screening.
